# Association of Past *Chlamydia trachomatis* Infection With Miscarriage

**DOI:** 10.1001/jamanetworkopen.2020.18799

**Published:** 2020-10-07

**Authors:** Andrew W. Horne, Nick Wheelhouse, Patrick J. Horner, W. Colin Duncan

**Affiliations:** 1 MRC Centre for Reproductive Health, Queen's Medical Research Institute, The University of Edinburgh, Edinburgh Bioquarter, Edinburgh, United Kingdom; 2 School of Applied Sciences, Edinburgh Napier University, Edinburgh, United Kingdom; 3 Population Health Sciences, University of Bristol, Bristol, United Kingdom

## Abstract

This case-control study assesses the association between past *Chlamydia trachomatis* infection with first-trimester miscarriage by using an enzyme-linked immunosorbent assay to detect antibodies to the protein Pgp3.

## Introduction

First-trimester miscarriages are commonly associated with chromosomal abnormality of the embryo (approximately 50% of cases).^1^ However, 15% of first-trimester and 66% of second-trimester miscarriages are attributed to reproductive tract infections.^2^ It has been suggested that *Chlamydia trachomatis* is a causative organism, but its association with miscarriage is inconsistently reported.^2,3,4^ This difference of opinion likely reflects the poor performance of major outer membrane protein (MOMP) peptide-based serology assays and the inability of nucleic acid amplification tests (which detect current infection) to detect previous exposure.^5^ It is now possible to accurately measure lifetime exposure to *C trachomatis* using an enzyme-linked immunosorbent assay (ELISA) that detects antibodies to the chlamydial plasmid-encoded protein Pgp3.^5^ This ELISA is more sensitive (73.8%) and specific (97.6%) than commercial ELISAs, including the Medac MOMP-peptide ELISA, or previous serological antibody tests.^5^ Pgp3 is unique to *C trachomatis*, eliminating cross-reactivity with antibodies to *C pneumoniae* infection (a common respiratory pathogen), a major weakness of previous serological tests. The aim of this study was to provide an estimation of the association of previous *C trachomatis* infection with the risk of spontaneous first-trimester miscarriage.

## Methods

We performed a case-control study, recruiting women with ultrasonography confirming absence of a fetal heart in the first trimester of pregnancy (miscarriage group) and women with normal pregnancies that had progressed into the third trimester (control group) from the same catchment population. Women with a past history of miscarriage were excluded from the control group. Participants were identified from the Pregnancy Support Unit and Delivery Suite at the Royal Infirmary of Edinburgh (a large UK National Health Service teaching hospital). The first study participant was recruited on January 22, 2013, and the last participant was recruited on September 26, 2019. The Scotland A Research Ethics Committee approved this study, and written informed consent was obtained from all participants. This study followed the Strengthening the Reporting of Observational Studies in Epidemiology (STROBE) reporting guideline.

We anticipated a *C trachomatis* seroprevalence of 15% in women with miscarriage and 7% in the control group on the basis of literature review^3^ and pilot work. Our proposed sample size (200 cases and 100 controls) had greater than 95% power, with a level of significance (α) of .05 to estimate a doubling of the *C trachomatis*–population attributable risk for miscarriage. We collected serum samples and self-taken vulvovaginal swabs taken from 2 to 3 inches within the vagina for *C trachomatis* nucleic acid amplification testing to detect current infection. Statistical analyses were conducted using GraphPad Prism, version 8.0 (GraphPad). Analysis was by 2-tailed Fisher exact test, and *P* < .05 indicated significance.

## Results

A total of 251 women (median [95% CI] age, 33 [32-35] years) were included in the miscarriage group, and 118 were included in the control group (median [95% CI] age, 34 [32-35] years). The groups were well balanced for all characteristics measured at baseline (Table). A total of 65 women (25.9%; 95% CI, 20.6%-31.4%) in the miscarriage group and 33 women (28.0%; 95% CI, 19.9%-36.1%) in the control group had positive test results for Pgp3 antibodies, suggesting previous infection with *C trachomatis* (*P* = .71). There was no evidence of active *C trachomatis* infection in either group. More women in the miscarriage group (n = 34 [13.5%; 95% CI, 11.3%-15.7%]) than the control group (n = 2 [1.7%; 95% CI, 0.5%-2.9%]) self-reported past *C trachomatis* infection (*P* < .001).

**Table.** Patient Characteristics

Characteristic	No. (%)		*P* value^a^
	Controls (n = 118)	Miscarriage group (n = 251)	
Age, median (95% CI), y	34 (32-35)	33 (32-35)	.72
BMI, mean (95% CI)	26.0 (25.0-27.0)	25.6 (24.9-26.3)	.55
Self-reported past *C trachomatis* infection, No. (%)	2 (1.7)	34 (13.5)	<.001
*C trachomatis* seropositivity	33 (28.0)	65 (25.9)	.71
Prior miscarriage	NA	106 (41.4)	NA
Prior live births	85 (72.0)	127 (50.6)	<.001
History of smoking			
Never	75 (66.4)^b^	165 (66.8)^c^	>.99
Ex-smoker	27 (23.9)^b^	60 (24.3)^c^	>.99
Smoker	11 (9.7)^b^	22 (8.9)^c^	.84

Abbreviations: BMI, body mass index (calculated as weight in kilograms divided by height in meters squared); *C trachomatis*, *Chlamydia trachomatis*; NA, not applicable.

^a^ *P* < .05 indicates statistical significance.

^b^ 113 responses.

^c^ 247 responses.

## Discussion

Contrary to the study by Baud et al,^3^ which was conducted on a similar-sized data set using a MOMP-peptide ELISA, the present study, using the more sensitive Pgp3 ELISA, found no significant association of past *C trachomatis* exposure with spontaneous first-trimester miscarriage. The lack of genetic analysis of the miscarriages and inability to match for past obstetric history are limitations of the study. It is unclear why more women in the miscarriage group self-reported *C trachomatis* infection, as recall bias is unlikely to explain such a difference. One possibility is that women in the miscarriage group were more likely to have had symptomatic *C trachomatis* infection and therefore seek testing. However, the seroprevalence rates of over 25% observed in both cohorts suggest that the prevalence of *C trachomatis* infection in young women—and the potential clinical outcomes of other reproductive disorders, such as female infertility and ectopic pregnancy—remain underestimated.
